# Enterohepatic bacterial infections dysregulate the FGF15-FGFR4 endocrine axis

**DOI:** 10.1186/1471-2180-13-238

**Published:** 2013-10-29

**Authors:** Guillaume Romain, Sarah Tremblay, Ellen T Arena, L Caetano M Antunes, Scott Covey, Michael T Chow, B Brett Finlay, Alfredo Menendez

**Affiliations:** 1 Department of Microbiology and Infectious Diseases, Faculty of Medicine and Health Sciences, University of Sherbrooke, Cancer Research Pavilion, Rm Z8-1072, 3201, rue Jean-Mignault, Sherbrooke, Québec J1E 4K8, Canada; 2 Michael Smith Laboratories, The University of British Columbia, Vancouver, BC V6T 1Z4, Canada; 3 Department of Biochemistry and Molecular Biology, The University of British Columbia, Vancouver, BC V6T 1Z3, Canada; 4 Department of Microbiology and Immunology, University of British Columbia, Vancouver, BC V6T 1Z3, Canada; 5 Present address: Unité de Pathogénie Microbienne Moléculaire Institut Pasteur, 28 rue du Dr Roux, F – 75724, Paris Cédex 15, France; 6 Present address: Escola Nacional de Saúde Pública Sergio Arouca, Fundação Oswaldo Cruz, Rua Leopoldo Bulhões, 1480, Rio de Janeiro, RJ 21041-210, Brazil; 7 Present address: Qu Biologics Inc, 887 Great Northern Way, Suite 138, Vancouver, BC V5T 4T5, Canada

**Keywords:** Endocrine, Metabolism, Enterohepatic, Infection, FGF15, FGF19, FGFR4, βKlotho, *Salmonella*, *Listeria*

## Abstract

**Background:**

Enterohepatic bacterial infections have the potential to affect multiple physiological processes of the body. Fibroblast growth factor 15/19 (FGF15 in mice, FGF19 in humans) is a hormone that functions as a central regulator of glucose, lipid and bile acid metabolism. FGF15/19 is produced in the intestine and exert its actions on the liver by signaling through the FGFR4-βKlotho receptor complex. Here, we examined the *in vivo* effects of enterohepatic bacterial infection over the FGF15 endocrine axis.

**Results:**

Infection triggered significant reductions in the intestinal expression of *Fgf15* and its hepatic receptor components (*Fgfr4* and *Klb* (βKlotho)). Infection also resulted in alterations of the expression pattern of genes involved in hepatobiliary function, marked reduction in gallbladder bile volumes and accumulation of hepatic cholesterol and triglycerides. The decrease in ileal *Fgf15* expression was associated with liver bacterial colonization and hepatobiliary pathophysiology rather than with direct intestinal bacterial pathogenesis.

**Conclusions:**

Bacterial pathogens of the enterohepatic system can disturb the homeostasis of the FGF15/19-FGFR4 endocrine axis. These results open up a possible link between FGF15/19-FGFR4 disruptions and the metabolic and nutritional disorders observed in infectious diseases.

## Background

Alteration of the host’s metabolism is common in infectious diseases; it can lead to patient malnutrition and the need for nutritional support [1,2]. Infection-driven metabolic changes are characterized by an accelerated flux of glucose, lipids, proteins and amino acids that may result in net protein loss and diabetic-like hyperglycemia [1,2]. Significant metabolic disorders have been observed in natural and experimental infections with the bacterium *Salmonella enterica*, including changes of the lipid and protein profiles and widespread hormonal imbalances [1,3,4]. In humans, *Salmonella enterica* serovar Typhi causes typhoid fever, a disease characterized by multi-organ bacterial colonization with common immunopathological manifestations in the gastrointestinal tract and the hepatobiliary system [5].

The molecular and physiological bases of the metabolic disorders observed during infection are not fully understood. In this work, we examined the disruption of the enterohepatic fibroblast growth factor 15/19 (FGF15/19)-fibroblast growth factor receptor 4 (FGFR4) endocrine axis during bacterial infections of the enterohepatic system. FGF15/19 (FGF15 in mice, FGF19 in humans) is an endocrine factor secreted by intestinal enterocytes [6]. FGF15/19 has a crucial role in the control of whole body glucose and lipid metabolism and energy expenditure [7,8]. It is also a key regulator of de novo synthesis of bile acids via the repression of cholesterol 7 alpha hydroxylase (CYP7A1) expression in hepatocytes [9]. In addition, FGF15 represses the apical Na^+^-dependent bile acid transporter (ASBT) expression in hepatic cholangiocytes [10] and facilitates gallbladder filling by promoting gallbladder muscle distension [11]. Through these functions, FGF15/19 closes an important negative feedback loop in the regulation of bile acid homeostasis. Signaling to hepatic target cells occurs through the interaction of FGF15/19 with the tyrosine kinase receptor fibroblast growth factor receptor 4 (FGFR4) and also requires the protein βKlotho. Mice genetically deficient for *Fgf15*, *Fgfr4* or *Klb* (βKlotho) have similar biliary phenotypes with higher levels of CYP7A1 and increased synthesis of bile acids [6,12-14]. Reduced FGF19 levels have been observed in patients with inflammatory bowel disease [15] and chronic idiopathic bile acid diarrhea [16]. On the other hand, patients with insulin resistance and non-alcoholic fatty liver disease, as well as extrahepatic cholestasis frequently display elevated plasma levels of FGF19 [17,18].

Using a model of murine typhoid fever, we demonstrate that *Salmonella enterica* infection triggers major alterations in the hepatic biliary function gene expression program, promotes accumulation of hepatic cholesterol and triglycerides and leads to a significant reduction in physiological gallbladder bile volumes. In addition, *Salmonella* infection causes a substantial decrease in the expression of intestinal *Fgf15*, accompanied by a dramatic loss of hepatic FGFR4 and βKlotho. These disturbances appear to be secondary to hepatic inflammation. Given the important role of the FGF15/19-FGFR4 endocrine axis as a central metabolic regulator, these alterations may be a major factor underlying the pathophysiology of bacterial infectious diseases.

## Methods

### Bacterial strains and mouse infections

*Salmonella enterica* serovar Typhimurium strains SL1344 (Sm^r^) and SB103 (*invA*) [19] and *Listeria monocytogenes* 10403 s (Sm^r^) [20] were used in this study. Bacteria were grown overnight at 37°C in LB supplemented with 100 μg/mL streptomycin. Inoculum was prepared in sterile HEPES 100 mM, NaCl 0.9%, pH 8.0. Animal protocols were approved by the Animal Care Committees of the University of British Columbia and the University of Sherbrooke. Eight weeks-old female C57BL/6 mice (The Jackson Laboratory, Bar Harbor, USA) were infected orally with 5 × 10^7^*Salmonella* SL1344, intravenously with 5 × 10^2^*Salmonella* SB103 or with *Listeria* 10403 s (2 × 10^9^ bacteria orally and 10^4^ intravenously). The animals were kept with food and water *ad libitum* through the duration of the study and were always sacrificed during the light period (10:00 AM ± 60 minutes). The bile was collected by gallbladder resection and draining by puncture. For bacterial counts, tissues were homogenized using a Mixer Mill MM400 (Retsch GmbH) followed by plating of serial dilutions in LB plates containing 100 μg/mL streptomycin. All infection experiments were done in duplicate using a total of 8–10 mice per group.

### Expression analyses

Ileum and liver samples were collected for mRNA and protein analysis. The ileal samples were taken approximately 2 cm away from the ileo-cecal junction; liver samples were taken from the central lobule. RNA was extracted using the RNeasy kit (Qiagen) and cDNA was prepared using the Quantitech Reverse Transcription kit (Qiagen). Quantitative PCR (qPCR) were done on an Eppendorf RealPlex^2^ system using the DyNamo SYBR Green qPCR Kit (Thermo Scientific). All reactions were done in 10 μl final volume with 40 cycles of 30 seconds denaturing at 95°C, 30 seconds annealing at 60°C and 30 seconds extension at 72°C (except the annealing temperature for Ostβ: 62°C). The relative expressions were calculated using the ddCt method and corrected for primer efficiencies according to Pfaffl *et. al.*[21]. The qPCR primers are listed in Table 1. Western blots were performed using total liver tissue lysates and antibodies against CYP7A1 (Abcam, ab65596, 1:1000), FGFR4 (Abcam, ab119378, 1:500), βKlotho (R&D, AF2619, 1:2000) and actin (SIGMA A4700, 1:1000).

**Table 1:** The genes analyzed in this study and the sequences of the qPCR primer sets

**Gene**	**Official symbol**	**Product**	**Primers**
Abcg5	*Abcg5*	ATP-binding cassette, sub-family G (WHITE), member 5	TGTCAACAGTATAGTGGCTCTG
			CGTAAAACTCATTGACCACGAG
Abcg8	*Abcg8*	ATP-binding cassette, sub-family G (WHITE), member 8	CTTGTCCTCGCTATAGCAACC
			TTTCCACAGAAAGTCATCAAAGC
Asbt	*Slc10a2*	Apical sodium-dependent bile acid transporter	ACCTTCCCACTCATCTATACTG
			CAAATGATGGCCTGGAGTCC
Bsep	*Abcb11*	Bile salt export pump	CAACGCATTGCTATTGCTCGG
			TAGACAAGCGATGAGCAATGAC
Cyp7a1	*Cyp7a1*	Cholesterol 7 alpha hydroxylase	GGGAATGCCATTTACTTGGATC
			TATAGGAACCATCCTCAAGGTG
Fabp6	*Fabp6*	Fatty acid binding protein 6	GAATTACGATGAGTTCATGAAGC
			TTGCCAATGGTGAACTTGTTGC
Fgf15	*Fgf15*	Fibroblast growth factor 15	AGACGATTGCCATCAAGGACG
			GTACTGGTTGTAGCCTAAACAG
FgfR4	*Fgfr4*	Fibroblast growth factor receptor 4	CTCGATCCGCTTTGGGAATTC
			CAGGTCTGCCAAATCCTTGTC
FXR	*Nr1h4*	Farnesoid X receptor (nuclear receptor subfamily 1, group H, member 4)	GTTCGGCGGAGATTTTCAATAAG
			AGTCATTTTGAGTTCTCCAACAC
βKlotho	*Klb*	Beta Klotho	AACAGCTGTCTACACTGTGGG
			ATGGAGTGCTGGCAGTTGATC
Mdr1a	*Abcb1a*	ATP-binding cassette, sub-family B member 1a	CCGATAAAAGAGCCATGTTTGC
			CTTCTGCCTGATCTTGTGTATC
Mdr1b	*Abcb1b*	ATP-binding cassette sub-family B member 1b	GGACCCAACAGTACTCTGATC
			ACTTCTGCCTAATCTTGTGTATC
Mdr2	*Abcb4*	Multidrug resistance protein 2	TTGTCAATGCTAAATCCAGGAAG
			AGTTCAGTGGTGCCCTTGATG
Mrp2	*Abcc2*	ATP-binding cassette, sub-family C (CFTR/MRP) member 2	GGCTCATCTCAAATCCTTTGTG
			TTTTGGATTTTCGAAGCACGGC
Mrp3	*Abcc3*	ATP-binding cassette, sub-family C (CFTR/MRP), member 3	GAACACGTTCGTGAGCAGCC
			ATCCGTCTCCAAGTCAATGGC
Mrp4	*Abcc4*	ATP-binding cassette, sub-family C (CFTR/MRP), member 4	TACAAGATGGTTCAGCAACTGG
			GTCCATTGGAGGTGTTCATAAC
Ntcp	*Slc10a1*	Sodium-taurocholate co-transporting polypeptide	CGTCATGACACCACACTTACTG
			GATGGTAGAACAGAGTTGGACG
Osta	*Osta*	Organic solute transporter alpha	TCTCCATCTTGGCTAACAGTG
			GATAGTACATTCGTGTCAGCAC
Ostb	*Ostb*	Organic solute transporter beta	CCACAGTGCAGAGAAAGCTGC
			ACATGCTTGTCATGACCACCAG
Shp	*Nr0b2*	Small heterodimer partner	AGTCTTTCTGGAGCCTTGAGC
			TTGCAGGTGTGCGATGTGGC
SrbI	*Scarb1*	Scavenger receptor class B type 1	GAACTGTTCTGTGAAGATGCAG
			GCGTGTAGAACGTGCTCAGG
36B4	*Rplp0*	Ribosomal protein, large, P0	TCTGGAGGGTGTCCGCAAC
			CTTGACCTTTTCAGTAAGTGG

The top sequence of each set corresponds to the forward primer and the bottom one to the reverse. All reactions were done in 10 μl final volume with 40 cycles of 30 seconds denaturing at 95°C, 30 seconds annealing at 60°C and 30 seconds extension at 72°C (except annealing temperature for Ostβ, which was 62°C).

### Microscopy

For histological analysis, tissue sections were fixed in 10% buffered formalin, embedded in paraffin and stained with H&E. Alternatively, samples fixed in 3.5% paraformaldehyde and frozen-embedded in OCT were used for immunofluorescent microscopy as previously described [22]. Fluorescence was visualized using an Olympus IX81 microscope.

### Cholesterol and triglyceride determinations

Cholesterol and triglycerides were assayed in liver lysates. A total of 40-100 mg of liver was homogenized with an ultra turrax (setting 5, 4 times for 15 sec) in 3 ml of chloroform:methanol (2:1), extracted twice with water, and centrifuged for 15 minutes at 3000 *g*. For the triglyceride assay 200 μl of the organic layer (lower phase) was removed and evaporated under N_2_(*g*). 10 μl of Thesit (Sigma-Aldrich, St Louis, MO) was added and mixed under N_2_(*g*). Water (50 μl) was added and incubated at 37°C for 1 hr with intermittent vortexing. Aliquots of 5 μl were assayed using the Serum Triglyceride Determination kit (Sigma-Aldrich, St Louis, MO) modified for a 96-well plate, calibrated with a trioleate (Sigma-Aldrich, St Louis, MO) standard curve. The cholesterol assay was performed at the same time but 500 μl of the organic layer (lower phase) was removed after the centrifugation step and evaporated under N_2_(*g*). 50 μl of isopropanol was then added to the dried down lipids and mixed by vortexing. Aliquots of 2 μl were then assayed using the Cholesterol E kit (Wako Chemicals USA, Richmond, USA).

### Statistical analyses

Data processing and statistical analyses were performed using GraphPad Prism5. Student’s *t* test was applied to all sets of data for statistical comparisons between groups, the graphs show the means and the standard errors of the mean.

## Results

### Enterohepatic infections downregulate the expression of intestinal *Fgf15*

**Figure 1:**
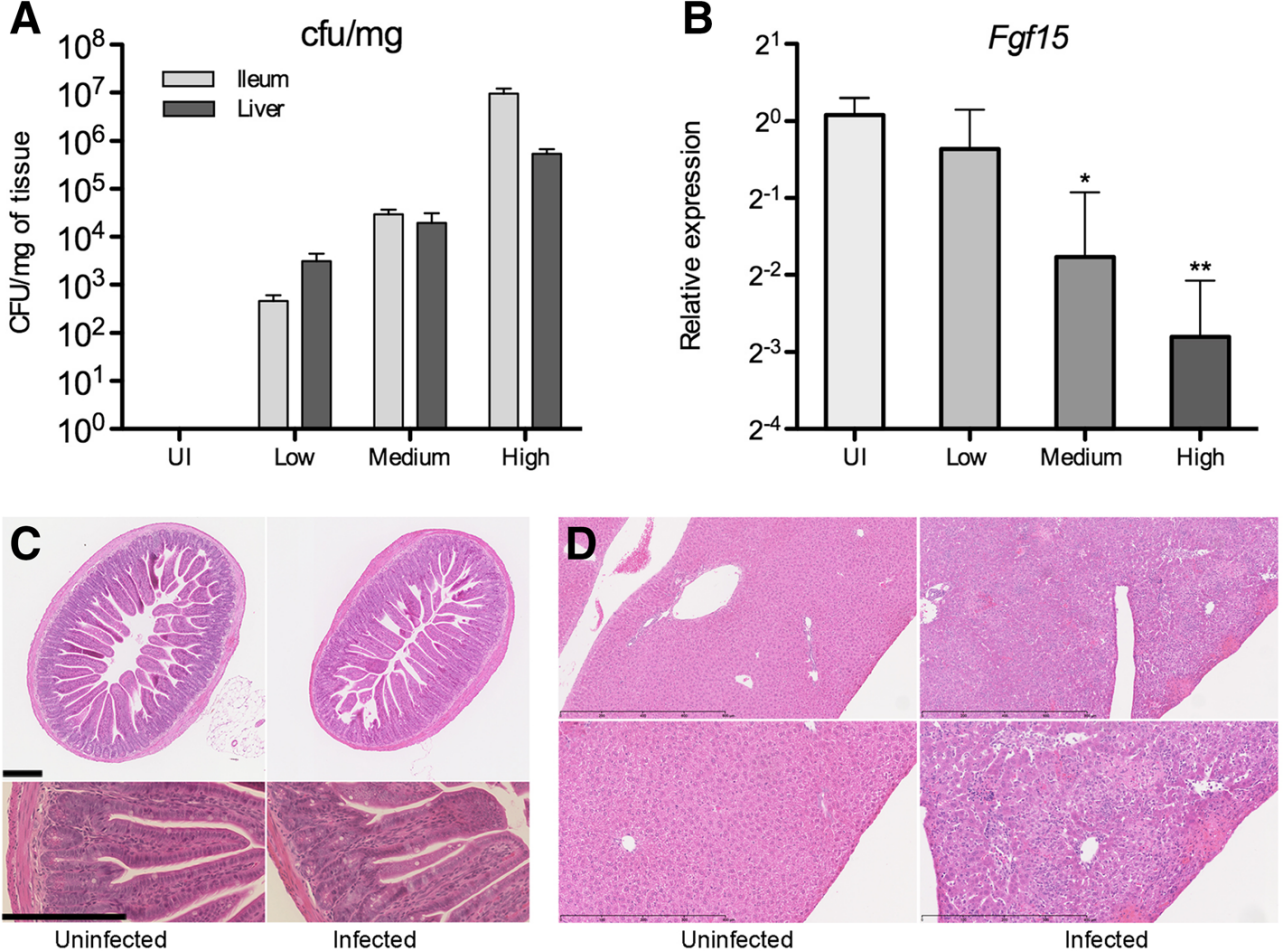
**Oral infection with *Salmonella typhimurium *SL1344 decreases the expression of *Fgf15 *in the ileum. (A)** bacterial counts in infected ilea and livers; animals were arbitrarily grouped into low, medium and high infection levels (10^0^-10^3^, 10^4^-10^5^ and >10^6^ cfu/mg, respectively roughly corresponding to 72, 96 and 120 hours post-infection; UI: uninfected). **(B)** relative levels of *Fgf15* transcripts in the ilea of infected mice (data by qPCR). **(C)** H&E staining of ileum sections from representative uninfected and orally *Salmonella*-infected animals (ileal colonization of the infected animal = 2.2 × 10^6^ cfu/mg); scale bars are 200 μm. **(D)** H&E staining of liver sections from representative uninfected and orally *Salmonella*-infected animals (liver colonization of the infected animal = 1.7 × 10^5^ cfu/mg); scale bars are 800 and 400 μm.

The terminal ileum is the main site of production of FGF15, it is also a major port of entry for *Salmonella* and therefore, an important site for its pathogenesis. To determine the effect of *Salmonella* infection on the homeostatic synthesis of FGF15, we collected tissue samples from infected animals and analyzed the abundance of *Fgf15* transcripts by qPCR. As shown in Figures 1A and 1B, the level of *Fgf15* transcripts inversely correlated with bacterial counts in the liver and the ileum, with a statistically significant decrease observed at mid-high infection levels. While H&E-stained sections from the ileum of infected animals did not show signs of pathological alteration (Figure 1C), staining of liver sections demonstrated a strong inflammatory response evidenced by large lesions with widespread lymphocytic infiltration, extensive necrosis often accompanied by local hemorrhage, and zones of parenchymal degeneration characterized by disappearance of hepatocytes (Figure 1D).

**Figure 2:**
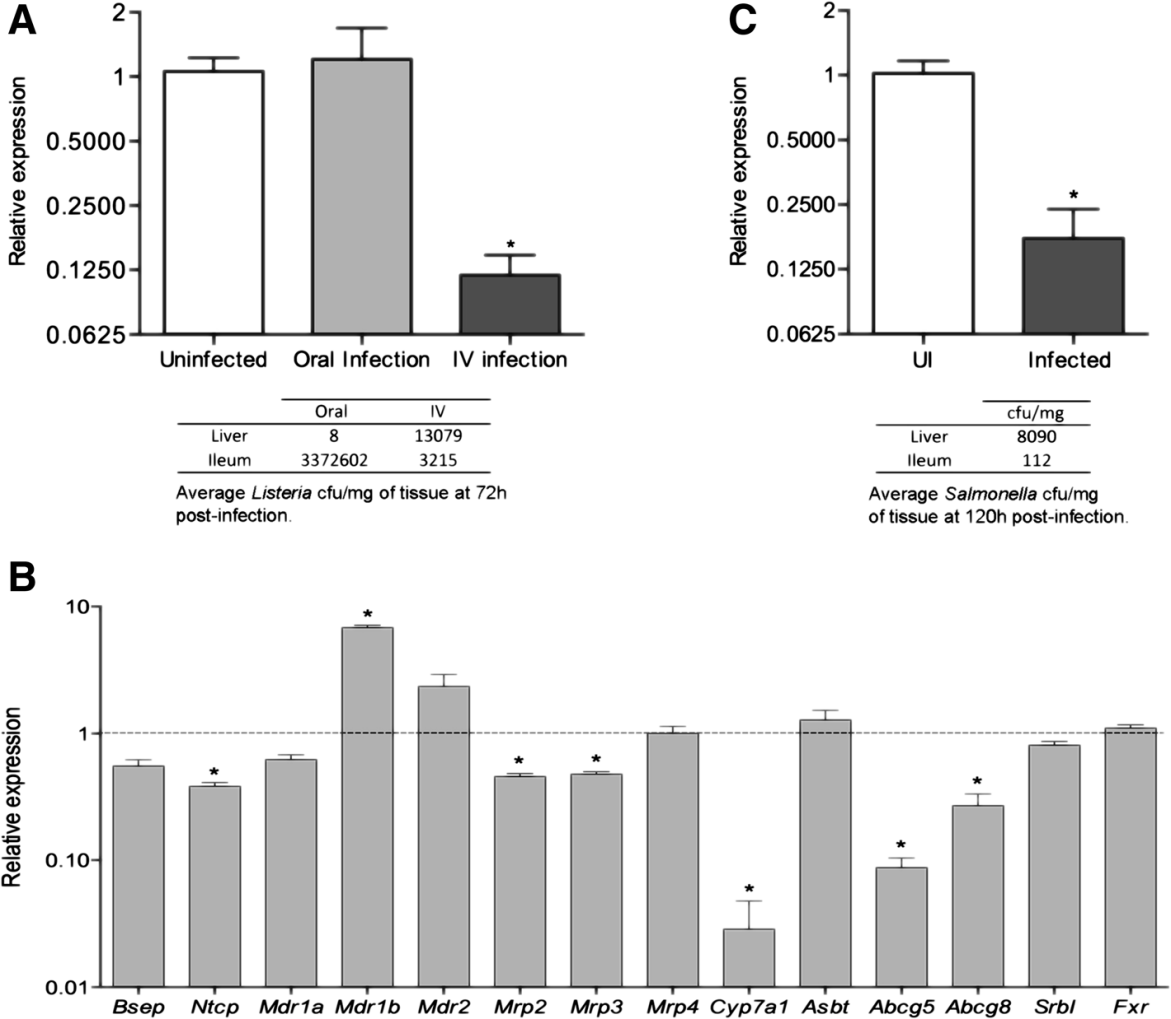
**Liver colonization drives the downregulation of ileal *Fgf15 *expression. (A)** relative levels of *Fgf15* transcripts in the ileum of mice infected orally or intravenously with *Listeria monocytogenes*. **(B)** transcript levels of genes involved in liver biliary metabolism in mice infected intravenously with *Listeria monocytogenes*, relative to the levels of uninfected animals (defined as 1, dashed line). **(C)** relative levels of *Fgf15* transcripts in the ilea of mice infected intravenously with *Salmonella typhimurium* SB103 (*invA*), at 120 hours post-infection. Data by qPCR, *p < 0.05.

FGF15 is synthesized by enterocytes [6], which can also be invaded by *Salmonella*[23]. However, the decrease in *Fgf15* expression was not associated with damage to the ileal enterocyte layer (Figure 1C). This suggests that loss of ileal enterocytes is not the reason for reduced *Fgf15* transcript levels. Oral infections with *Listeria monocytogenes*, an inefficient invader of the mouse intestinal epithelium [24,25], showed no significant liver colonization and large numbers of intestinal bacteria but not downregulation of *Fgf15* expression (Figure 2A). In contrast, intravenous infections with *Listeria*, which colonized the liver rapidly and triggered deccreases in the transcript levels of biliary function genes (Figure 2B), caused a significant reduction in ileal *Fgf15* expression (Figure 2A). These results point to hepatic pathophysiology, rather than intestinal bacterial colonization, as the primary event driving downregulation of intestinal *Fgf15* expression.

To establish the role of hepatic colonization and to probe the involvement of bacterial enterocyte invasion in repressing *Fgf15* expression, we carried out intravenous infections with the *Salmonella* invasion-deficient strain SB103 following Menendez *et al.*[22]. In this type of infection, *Salmonella* colonization of the hepatobiliary system occurs immediately whereas colonization of the gut is delayed by 72 to 96 hours [22]. Furthermore, the bacteria that eventually reach the intestines are unable to invade the enterocytes due to the *invA* mutation of this strain. As shown in Figure 2C, intravenous infection with *Salmonella* SB103 caused a reduction of *Fgf15* transcripts abundance. Notably, such a decrease was observed with a much lower intestinal bacterial burden than those in oral infections with the wild-type strain (average 10^2^*vs.* 10^7^ cfu/mg, respectively). These results demonstrate that colonization of the hepatobiliary system by *Salmonella* represses the expression of intestinal *Fgf15* and show that enterocyte invasion by intestinal bacteria does not play a major role on this effect.

**Figure 3:**
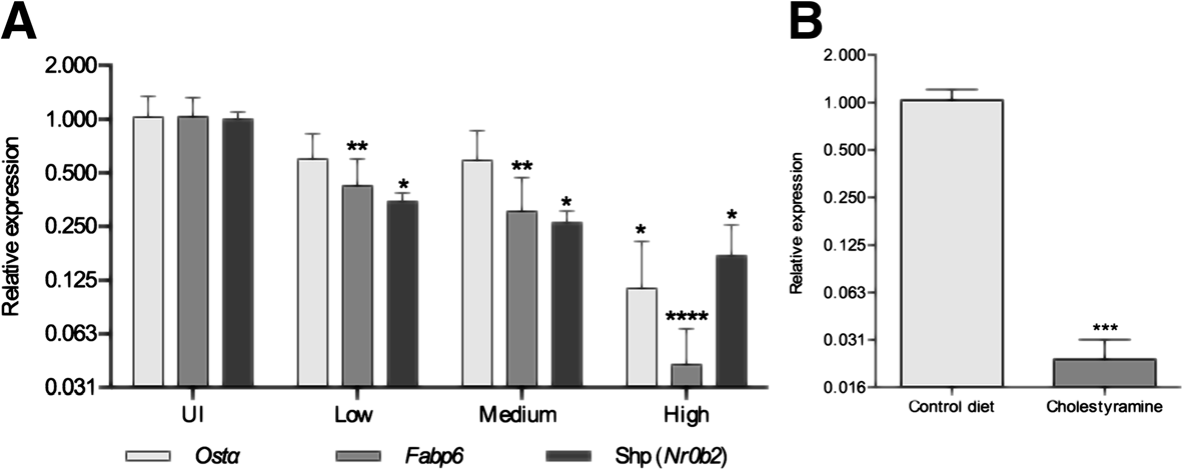
**Infection with *Salmonella *decreases the expression of *FXR-target genes *in the ileum. (A)** Relative levels of *Fabp6, Nr0b2* and *Osta* transcripts in the ileum of mice orally infected with *Salmonella typhimurium* SL1344. Animals were arbitrarily grouped into low, medium and high infection levels (10^0^-10^3^, 10^4^-10^5^ and >10^6^ cfu/mg, respectively roughly corresponding to 72, 96 and 120 hours post-infection; UI: uninfected). **(B)***Fgf15* transcript levels in the ilea of uninfected mice fed 5% cholestyramine diet. Data by qPCR, **p < 0.01; ***p < 0.001; ****p < 0.0001.

Transcription of *Fgf15* in ileal enterocytes is trans-activated by the nuclear receptor FXR (Farnesoid X Receptor), upon its activation by bile acids [7]. Expression of the FXR gene (*Nr1h4*) was not affected by *Salmonella*, regardless of the intestinal bacterial burden (data not shown). In contrast, the expression of other known intestinal FXR target genes, *Fabp6* (Fatty acid binding protein 6), *Nr0b2* (Small heterodimer partner, Shp) [26] and *Osta* (Organic solute transporter alpha) [27], was decreased by *Salmonella* infection in a pattern similar to that of *Fgf15* with maximal, significant drops in highly-infected animals (Figure 3A). This suggests that activation of gene expression mediated by FXR is impaired during infection.

Colonization of the hepatobiliary system by *Salmonella* induces local pathological damage and inflammation [22], which can result in impaired synthesis of bile acids and inflammation-induced cholestasis [28]. This may in turn, compromise intestinal FXR activation and lead to inhibition of *Fgf15*, *Fabp6*, *Nr0b2* and *Osta* expression. To test whether the depletion of bile acids would be sufficient to decrease *Fgf15* expression *in vivo*, we fed uninfected C57BL/6 mice with a diet supplemented with the bile acid sequestrant cholestyramine. As shown in Figure 3B mice fed with cholestyramine did have significantly lower levels of *Fgf15* transcripts than mice fed with a normal diet.

**Figure 4:**
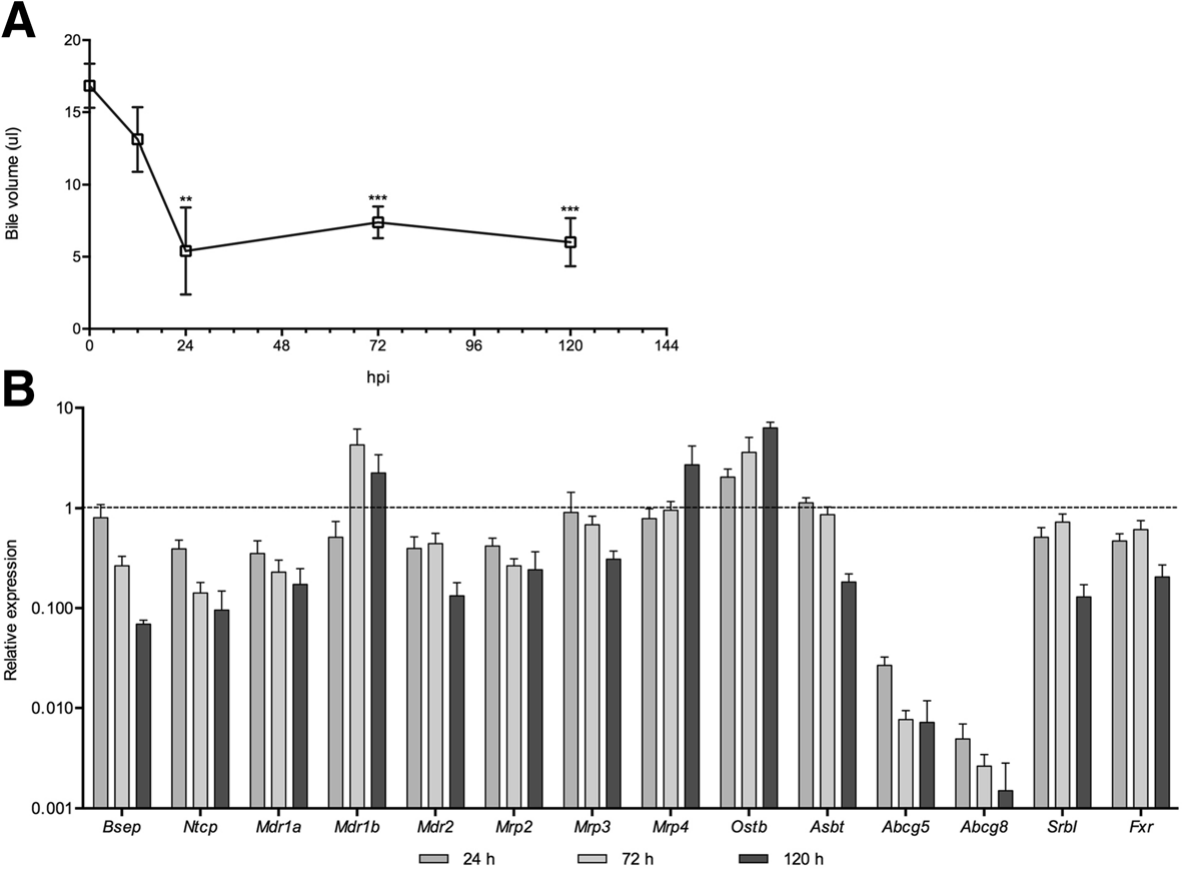
***Salmonella *infection perturbs the host’s hepatobiliary homeostasis. (A)** bile volumes recovered from the gallbladders of mice orally infected with *Salmonella* at the indicated hours post-infection (hpi). **(B)** Transcript levels of hepatic genes involved in liver biliary metabolism in mice infected with *Salmonella*, relative to the levels of uninfected animals (defined as 1, dashed line) at 24, 72 and 120 hours post-infection. Data by qPCR.

**Figure 5:**
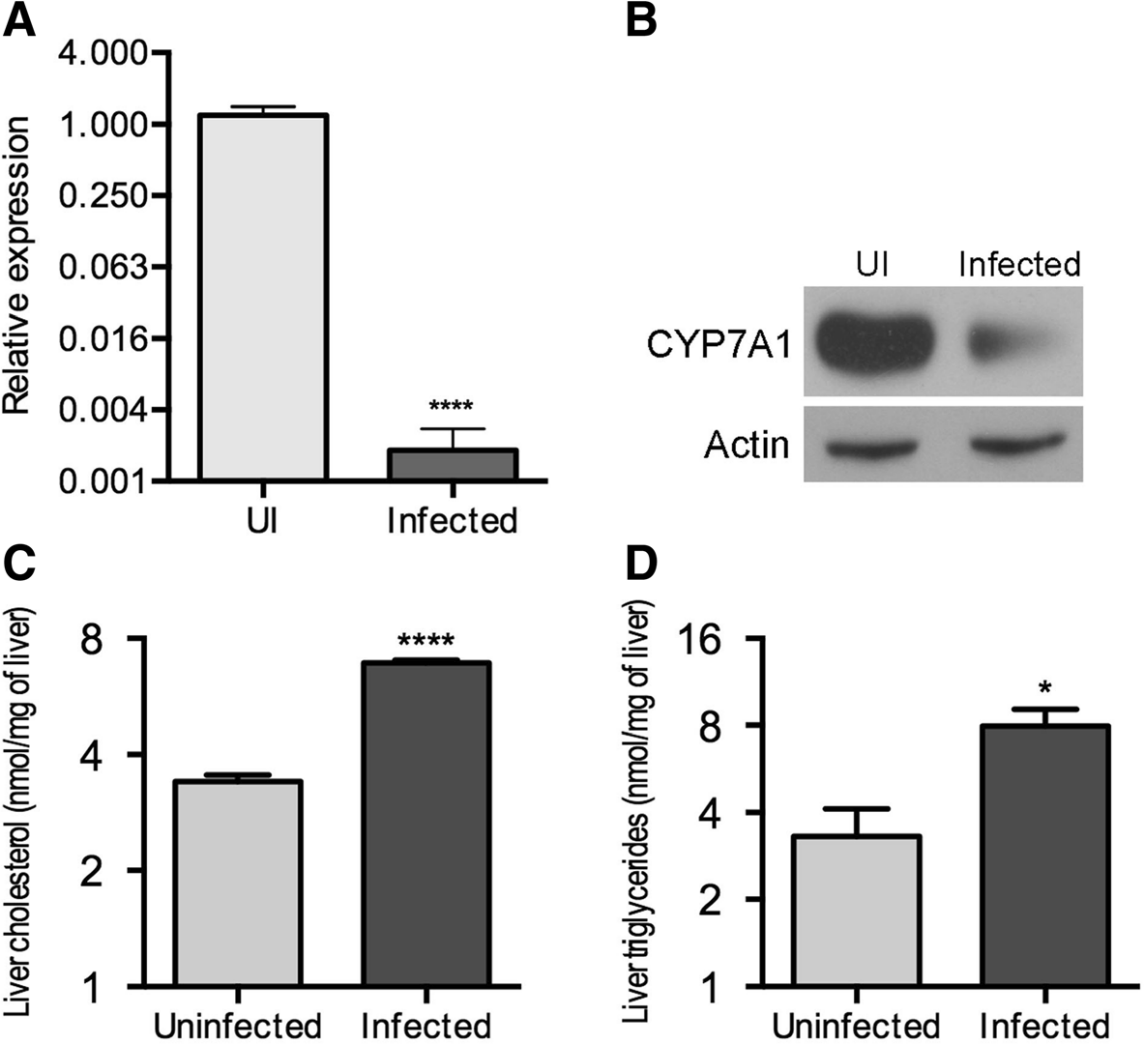
***Salmonella *infection downregulates the neutral bile acid synthesis pathway. (A)** relative levels of liver *Cyp7a1* transcripts in mice infected with *Salmonella*. **(B)** CYP7A1 western blot of liver lysates. **(C)** Cholesterol and **(D)** triglycerides accumulation in the liver of *Salmonella*-infected *vs.* uninfected mice, (*p < 0.05; ****p < 0.0001).

Second, we evaluated the effects of *Salmonella* infection in bile production and flow. Gallbladder bile volumes were measured before and during infection; a significant reduction in volume was observed 24 hours post-infection, which did not improved over the next 4 days (Figure 4A). An expression analysis of hepatic genes involved in bile synthesis and secretion (Figure 4B), showed striking reductions in the transcript levels of the major transporters of bile acid and cholesterol (*Abcb11*, *Slc10a1*, *Abcb1a*, *Abcg5* and *Abcg8*) and the induction of several genes involved in rescue from cholestasis. The mRNA (Figure 5A) and protein levels (Figure 5B) of CYP7A1, the rate-limiting enzyme in the neutral pathway of bile acids synthesis, were decreased by infection. This was accompanied by a significant accumulation of hepatic cholesterol and triglycerides (Figure 5C and Figure 5D), which collectively suggest interruption of bile synthesis and flow.

### *Salmonella* infection leads to depletion of the hepatic FGF15 receptor complex

**Figure 6:**
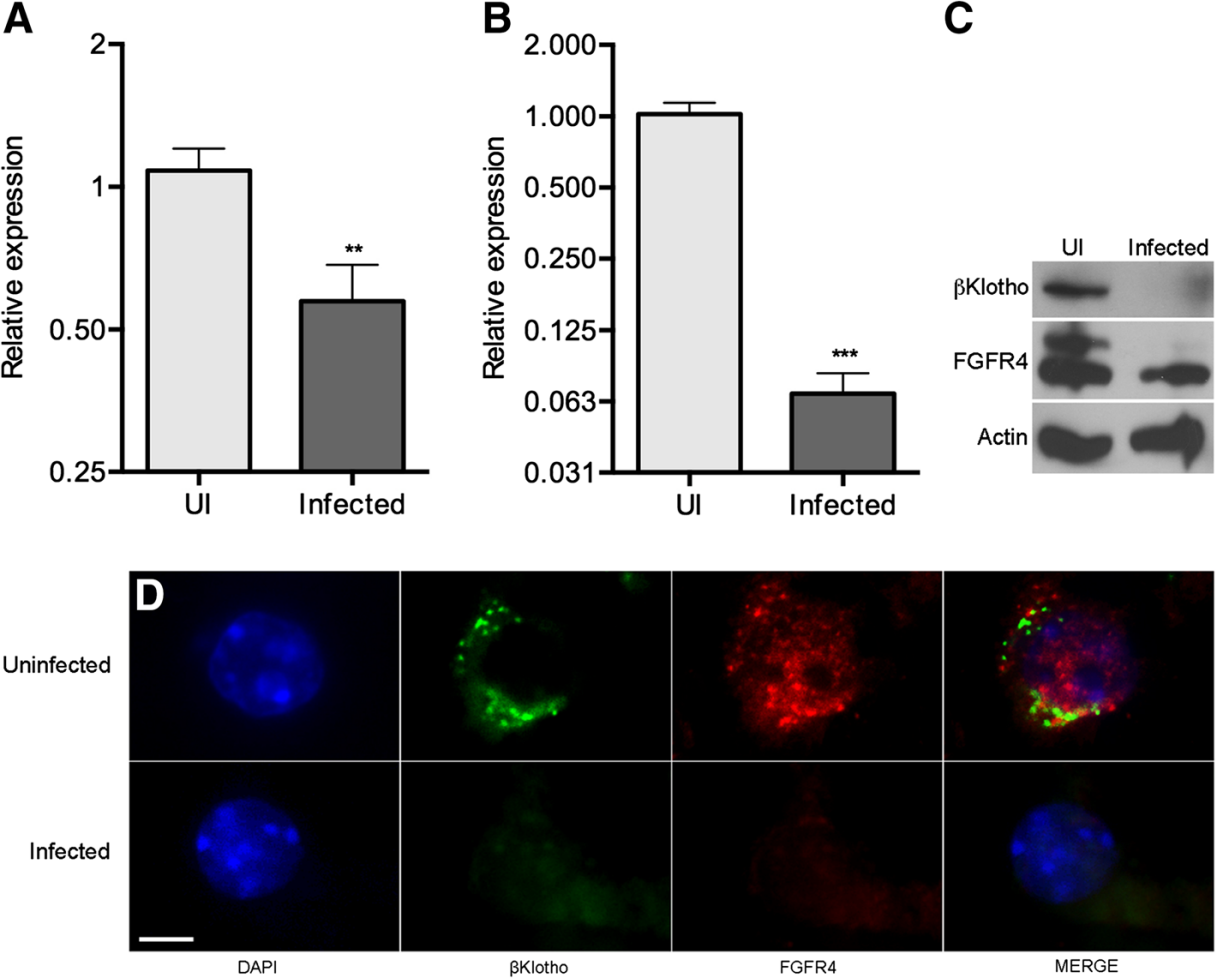
***Salmonella *infection causes the loss of the hepatic FGF15 receptor complex. (A)** relative levels of *Fgfr4* and **(B)***Klb* (βKlotho) transcripts in the livers of mice infected with *Salmonella*. The animals analyzed in **(A)** and **(B)** are from the high-infection group in Figure 1, the data is by qPCR, (**p < 0.01; ***p < 0.001). **(C)** FGFR4 and βKlotho western blots of liver lysates. **(D)** FGFR4 and βKlotho immunostaining of uninfected (top panel) and *Salmonella*-infected (bottom panel) liver samples. The figure shows a single, representative hepatocyte in each case. Scale bar is 5 μm.

Signaling of FGF15 in hepatocytes requires the tyrosine kinase membrane receptor FGFR4 and the protein βKlotho. To determine if *Salmonella* infection disturbs the homeostasis of this pathway, we analyzed the levels of FGFR4 and βKlotho in infected and uninfected livers. Figures 6A and 6B show that the transcript levels of both *Fgfr4* and *Klb* (βKlotho) were significantly decreased by infection. In addition, the protein levels were also reduced, as evidenced by western blot (Figure 6C). Two major FGFR4 bands were detected in uninfected animals, with apparent molecular weights of 115 and 125 KDa, likely corresponding to the core-glycosylated (FGFR4_115_) and fully-glycosylated, functional (FGFR4_125_) forms of FGFR4, respectively [29]. Infection led to the disappearance of FGFR4_125_ and a decrease of FGFR4_115_. Immunofluorescent staining of liver sections confirmed the reduction of FGFR4 and βKlotho. Both proteins were clearly detected in uninfected hepatocytes (Figure 6D); in contrast, hepatocytes from *Salmonella*-infected livers were devoid of FGFR4 and βKlotho.

## Discussion

The FGF19-FGFR4 endocrine axis is currently considered a potential intervention point for the therapy of cancer, gallstone disease, and metabolic disorders associated to the metabolic syndrome [7,30]. Experimental administrations of FGF19 and transgenic FGF19 mice have shown decreased liver fat content, improved hepatic and serum lipid profiles, and resistance to high-fat diet-induced obesity [31-33]. In addition, FGF15/19 induces hepatocyte proliferation [34] and has been recently identified as an important mediator of liver regeneration after liver resection surgery [35]. Here we show that *Salmonella* infection disturbs the homeostasis of the FGF15/19-FGFR4 axis by down-regulating the expression of *Fgf15, Fgfr4* and *Klb*. To our knowledge, these results constitute the first demonstration of a pathophysiological effect of bacterial infections over the FGF15/19-FGFR4 endocrine axis.

Infection modified both the ileal expression of *Fgf15* and the components of its hepatic receptor, which suggests a significant functional shutdown of the pathway. Our data rules out a direct cytopathic effect of bacteria over ileal enterocytes as the major cause of *Fgf15* mRNA reductions. Instead, it is apparent that the decline in *Fgf15* expression results from impaired activation of FXR in the enterocytes. Our interpretation is strongly supported by the observed low volumes of gallbladder bile and the decreased expression of *Fabp6, Ostα* and *Nr0b2* (Shp), all well-known FXR targets. In addition, we show that the depletion of the intestinal bile acids pool by oral administration of the bile acid sequestrant cholestyramine is sufficient to significantly decrease ileal *Fgf15* expression. Furthermore, intravenous infections with a *Salmonella* invasion mutant and with *Listeria monocytogenes*, both resulting in rapid hepatic colonization and pathophysiology, lead to reductions in *Fgf15* expression in the absence of significant ileal bacterial colonization or enterocyte invasion.

*Salmonella* infection induced a massive alteration of the hepatobiliary gene expression program. Remarkably, the mRNA and protein levels of CYP7A1, the rate-limiting enzyme in the neutral pathway of bile acids synthesis were decreased during infection, in spite of the lower levels of FGF15 which would be expected to promote the upregulation of *Cyp7a1* expression. These results reveal the complexities in the regulation of *Cyp7a1* expression and indicates that the mechanisms of *Cyp7a1* expression control are hierarchical. Infection also triggered a significant reduction of FGFR4 and βKlotho, the two proteins involved in assembling the functional receptor for FGF15 in hepatocytes. The biology of FGFR4 and βKlotho had never before been studied in the context of a bacterial insult, and our data suggest that their function can be severely compromised by bacterial infections *in vivo*. The mechanisms underlying their downregulation are unclear at present but we anticipate that they are related to the pro-inflammatory cytokine burst that follows liver colonization by bacteria. It has been recently reported that TNFα represses βKlotho expression in adipocytes [36]; thus it is possible that a similar mechanism acts in hepatocytes.

It is apparent that the dysregulation of the FGF15/19-FGFR4 endocrine axis components is not a general pathogenic feature of all bacteria, as infections with the enteric pathogen *Citrobacter rodentium*, the mouse model for human EPEC and EHEC [37], did not modify the expression of ileal *Fgf15* (data not shown). Instead, this pathophysiological effect may be restricted to infections displaying a relevant liver involvement. Further work is still necessary to define the full impact of infections in FGF15/19 function and to determine the underlying molecular mechanisms.

## Conclusions

Through the alteration of the hepatobiliary function, bacterial pathogens of the enterohepatic system dysregulate the homeostasis of the FGF15/19-FGFR4 endocrine axis. These revealing findings have important implications for the understanding of the pathophysiology of microbial diseases. Disruption of the FGF15/19-FGFR4 pathway may be a contributing factor to the metabolic and nutritional disorders associated with infectious diseases.

## Competing interests

The authors declare that they have no competing interests.

## Authors’ contributions

GR, ST, ETA and LCMA carried out *Salmonella* infections. GR performed the gene expression analysis, western blots and immunofluorescent microscopy. SC and ETA performed the cholesterol and triglyceride determinations. MTC carried out the *Listeria* infections. BBF participated in the supervision of the study. GR and AM drafted the manuscript. AM conceived the study and supervised its design, coordination and execution. All authors read and approved the final manuscript.
